# Tunable nanophotonics

**DOI:** 10.1515/nanoph-2022-0385

**Published:** 2022-08-11

**Authors:** Juejun Hu, Arseniy I. Kuznetsov, Volker J. Sorger, Isabelle Staude

**Affiliations:** Department of Materials Science & Engineering, Massachusetts Institute of Technology, Cambridge, MA, USA; Institute of Materials Research and Engineering, A^*^STAR (Agency for Science, Technology and Research), Singapore, Singapore; Department of Electrical and Computer Engineering, George Washington University, Washington, DC, USA; Institute of Solid State Physics, Friedrich Schiller University Jena, Jena, Germany

Most optical elements – be it refractive lenses, optical coatings, or glass fibers – have their attributes fully defined once they are fabricated. Moving or swapping bulky parts often becomes the only resort to enable dynamic tuning of an optical system to adapt to changing needs, which however escalates the system size, weight, complexity, and cost. The emergence of nanophotonics offers an unprecedented opportunity to realize *tunable* photonic systems. Through locally applying electrical, thermal, optical, or magnetic stimuli to change material properties, the optical functions of a nanophotonic device can be efficiently modulated with high speed, minimal power consumption, enhanced ruggedness, all awhile offering an ultracompact footprint. This unique capability foresees the widespread adoption of tunable nanophotonics in analog computing [1], high-speed communications [2], optical spectroscopy [3], computational imaging [4], beam steering [5], display technologies [6], optical zoom [7], nonreciprocal photonics [8], and numerous other applications of the looming Industry-4.0 era.

This special issue on “*tunable nanophotonics*” surveys this rapidly evolving field with emphases on novel active tuning mechanisms as well as emerging applications of reconfigurable nanophotonic devices. In their perspective article, Thureja et al. offer their viewpoint on technical pathways toward a “universal metasurface” capable of on-demand dynamically reconfigurable wavefront control. Fan et al. review the state-of-the-art of tunable metamaterials, introducing both fundamental concepts and latest advances in active tuning strategies. Ferreira de Lima et al. discuss automated design and control methods in reconfigurable neuromorphic photonic processors to counter variations and disturbances to realize resonator weight banks suitable for practical deployment [9]. Lian et al. review photonic memory technologies and their applications toward in-memory computing [10]. Taghavi et al. provide an overview on electro-optic polymer modulators, highlighting the path towards their integration with silicon photonics platforms.

The special issue encompasses a cohort of original research articles on diverse classes of new tunable materials and exciting optical physics enabled by nanophotonic tuning. Heβler et al. introduce In_3_SbTe_2_ as a phase change material (PCM) that can switch between plasmonic and dielectric states with a sign change in permittivity, and exploit this characteristic to switch optical antennas from sharp dielectric to broad plasmonic resonances [11]. Abdollahramezani et al. present a design of a PCM-based metasurface array with pixel-level tuning capability. Izdebskaya et al. demonstrate magnetic field tuning of liquid crystals (LCs) which avoids the needs for pre-alignment or structured electrode fabrication [12], and Gorkunov et al. combine electric field and mechanical displacement to control anomalous refraction in LC metasurfaces [13]. The study by John et al. reveals tunable anisotropy in epitaxially-grown VO_2_ films whose optical properties can be reconfigured from birefringent to hyperbolic [14], and Mei et al. show that free carrier concentrations and phase transition characteristics of VO_2_ films can be effectively modulated through focused ion beam irradiation [15]. Wu et al. model a metasurface made of ZnO with tunable topological properties using active optical pumping [16]. Kalaev et al. investigate electrochemical modulation of optical properties of praseodymium doped ceria, which exhibits chameleon-like color changing properties [17]. Vorobeva et al. identify an intriguing negative photoresponse phenomenon in Ti_3_C_2_T_*x*_ MXene flakes, where the photocurrent decreases over time under illumination. Tian et al. realize thermal tuning of structure-color metasurfaces made of a halide perovskite which undergoes a phase transition near room temperature [18]. Archetti et al. design varifocal silicon metalens with diffraction-limited performance based on thermo-optic tuning [19]. Ryabov et al. propose an analytical model for nonlinear photothermal effects due to thermorefractive bistability as well as an optimized design for efficient nonlinear optical heating [20]. Karvounis et al. integrate piezoelectric nanobeams made of BaTiO_3_ on a metasurface membrane, and report electromechanical tuning of the metasurface optical response [21]. Gui et al. theoretically study an ultra-compact modulator with 100 GHz bandwidth taking advantage of free carrier injection in indium tin oxide (ITO) [22]. Heidari et al. experimentally demonstrate a graphene-based modulator with remarkable 60 GHz bandwidth and 2.25 fJ/bit energy efficiency [23]. Tamura et al. theorize a mechanism for electrically tunable self-pulsation in a graphene-on-silicon resonator device [24]. Gao et al. achieve broadband tuning of nonlinear wavelength conversion in a nanostructured chalcogenide glass layer within femtosecond time scale [25]. Khorashad et al. elucidate the dynamics of hot carrier generation in tunable gap-plasmon metasurface absorbers [26]. Shafirin et al. explore a scheme to increase the quality (Q) factor of a nonlinear metasurface device after excitation over time, allowing them to overcome the classical time-bandwidth limit [27].

On emerging applications empowered by tunable nanophotonic devices, Brückerhoff-Plückelmann et al. demonstrate a photonic tensor core for performing matrix vector multiplications based on PCMs [28]. Teo et al. design an all-optical perceptron capitalizing on the nonlinear response of PCM to ultrafast laser pulse to realize nonlinear activation [29]. Liao et al. report a matrix eigenvalue solver based on graphene/Si thermo-optic reconfigurable photonic neural networks [30]. Sun et al. and Ma et al. demonstrate tunable thermal emitters using VO_2_ and chalcogenide PCM, respectively [31,32]. Yang et al. apply an LC-infiltrated spin-selective chiral metasurface to realize metaholograms with multi-level tunable intensities [33]. Sedeh et al. develop a new method of attaining optical nonreciprocity via pure temporal modulation of a metasurface. Finally, An et al. leverage deep neural networks to facilitate facile design of a metasurface-embedded high-Q tunable filter for spaceborne imaging spectroscopy [34].

Finally, emerging optical sources and photodetectors also benefit from nanoscale structuring; Heidari et al., for instance, demonstrated a transverse cavity-coupled VCSEL with higher speed and output power than conventional designs [35], and we showed that slot-nanophotonic approaches combined with 2-D materials allow for record-high gain-bandwidth-product photodetectors, a new class of devices [36].

In sum, it is our hope that this special issue offers a timely overview of the dynamic and rapidly growing field of tunable nanophotonics, and will spur further research, development, and educational efforts in this area. We want to express our genuine gratitude to all the authors and reviewers for their contributions. We also would like to thank the Nanophotonics Publishing editor Dennis Couwenberg and publishing assistant Tara Dorrian for their constant support throughout the review and production processes.
